# Supplementary material for: Integrative omics framework for characterization of coral reef ecosystems from the Tara Pacific expedition
Source: Sci Data. 2023 Jun 1;10:326. doi: 10.1038/s41597-023-02204-0 (PMC10235121; doi:10.1038/s41597-023-02204-0)
Supplement: Supplementary file 1 — Supplementary Figures and Tables [file 41597_2023_2204_MOESM1_ESM.pdf]

Integrative omics framework for characterization of coral reef ecosystems from the *Tara Pacific* expedition

Supplementary Figures and Tables

Supplementary Figure 1: Multiplexing strategies for Metabarcoding experiments.

Supplementary Figure 2: Control quality workflow for Metagenomic and Metatranscriptomic sequences.

Supplementary Figure 3: Control quality workflow for Metabarcoding sequences.

Supplementary Figure 4: Control quality workflow for Metabarcoding negative control sequences.

Supplementary Figure 5: RNA samples classification depending on their Agilent BioAnalyzer profile.

Supplementary Figure 6: Global view of the statistics generated by the control quality pipeline on a subset of sequences of each sequencing file.

Supplementary Figure 7: Taxonomic assignment of a sequencing file.

Supplementary Figure 8: Final report on the comparison between a Metabarcoding sample and negative controls.

Supplementary Table 1: List of abbreviations used in this study.

Supplementary Table 2: Barcode Identifier (BID) sequences.

Supplementary Table 3: PCR mixtures for Metabarcoding experiments.

Supplementary Table 4: PCR amplification cycling protocols for Metabarcoding experiments.

Supplementary Figure 1: Multiplexing strategies for Metabarcoding experiments. The multiplexing including BIDs allowed pooling of 6 to 12 PCR products in the same sequencing library.

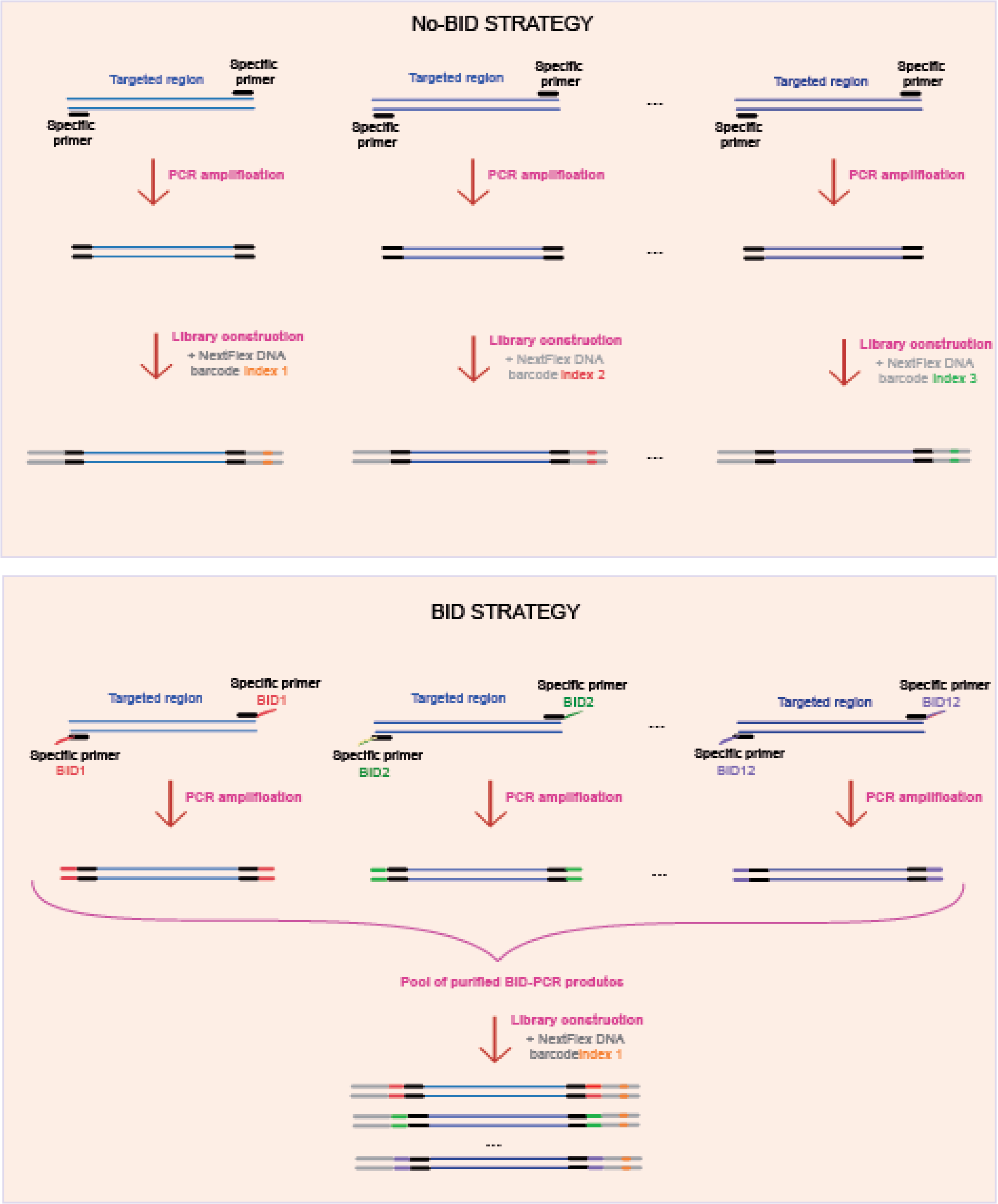

Supplementary Figure 2: Control quality workflow for Metagenomic and Metatranscriptomic sequences.

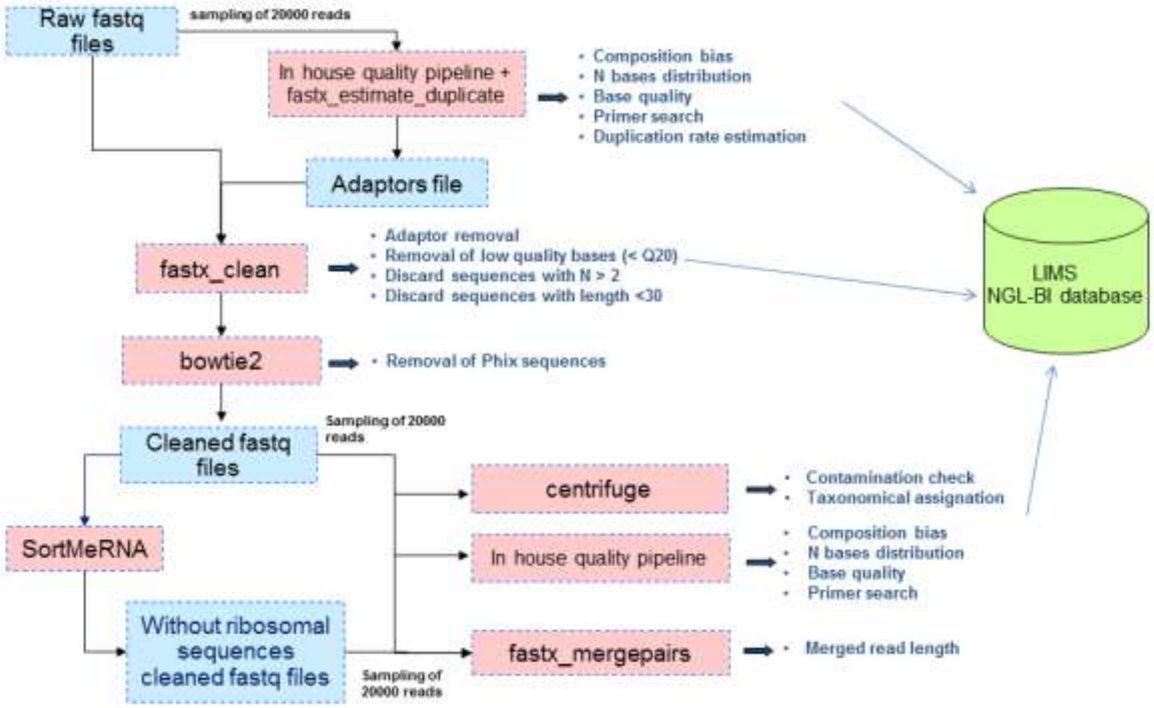

Supplementary Figure 3: Control quality workflow for Metabarcoding sequences.

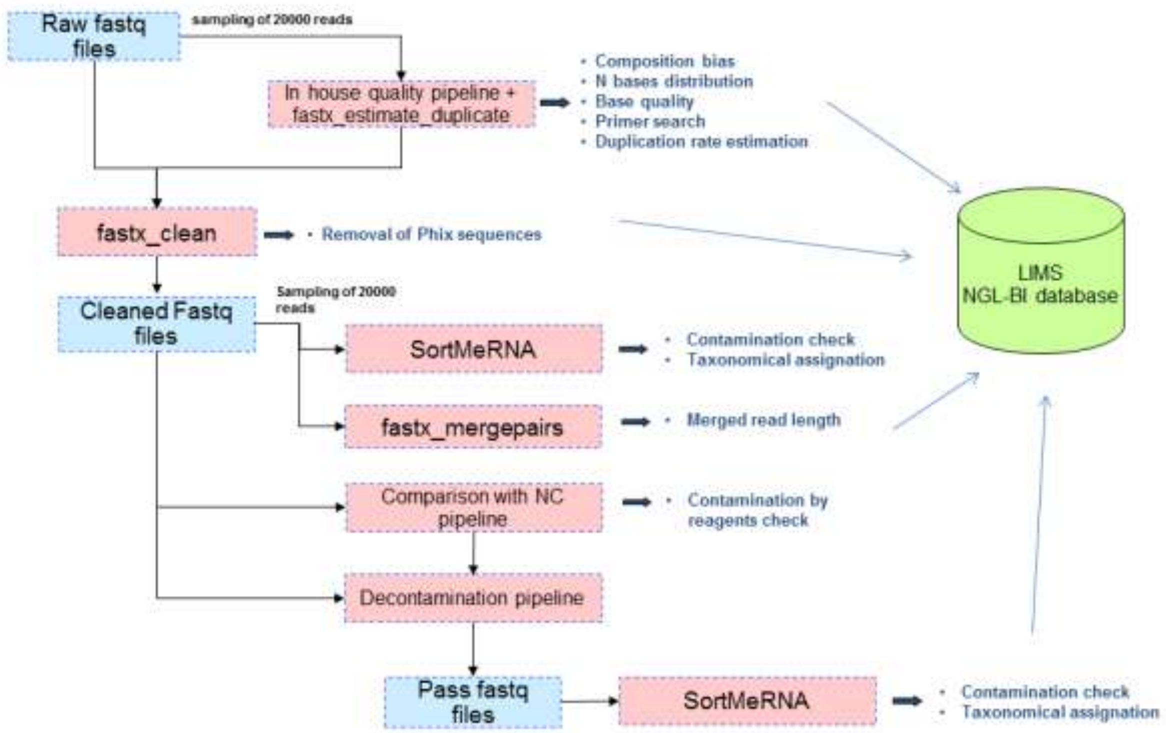

Supplementary Figure 4: Control quality workflow for Metabarcoding negative control sequences.

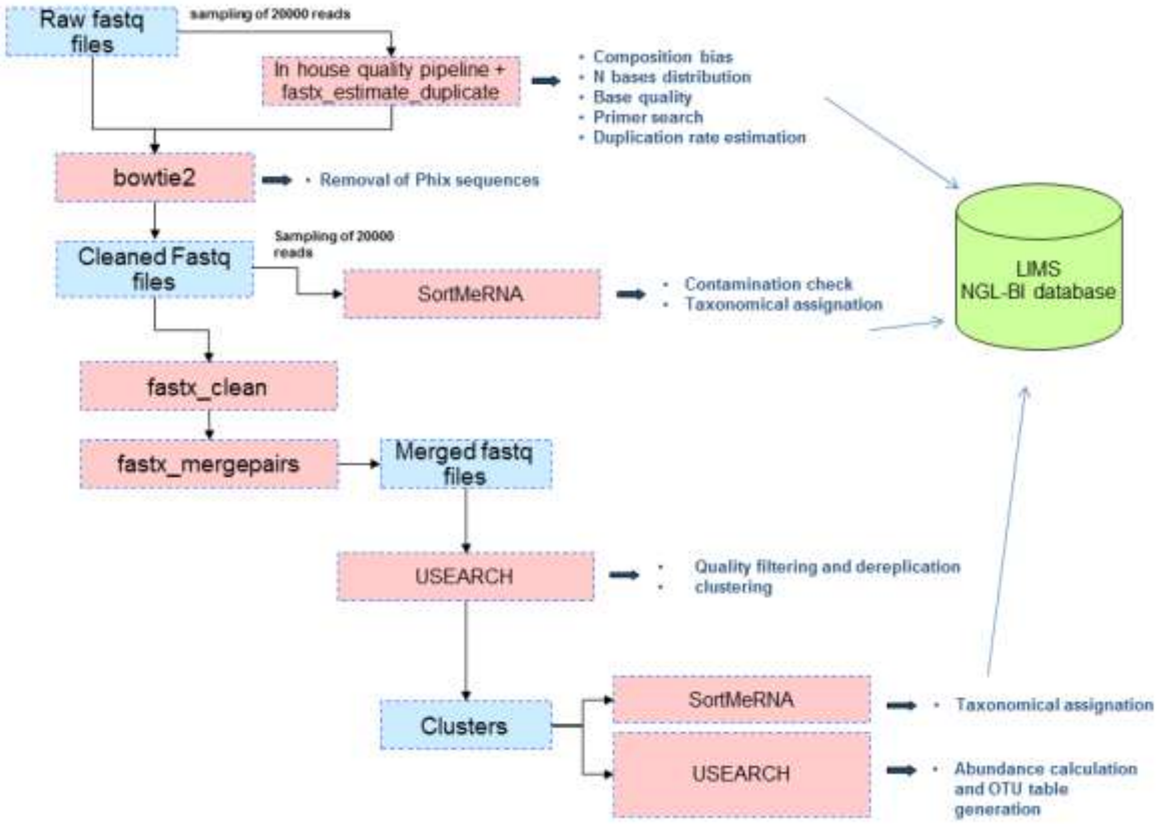

Supplementary Figure 5: RNA samples classification depending on their Agilent BioAnalyzer profile. Coral RNA samples exhibit different profiles: green profiles show high integrity of RNA, yellow and orange profiles exhibit rRNA peaks, but also variable amounts of small sized RNA and the red profile a rather comprehensive degradation of RNA.

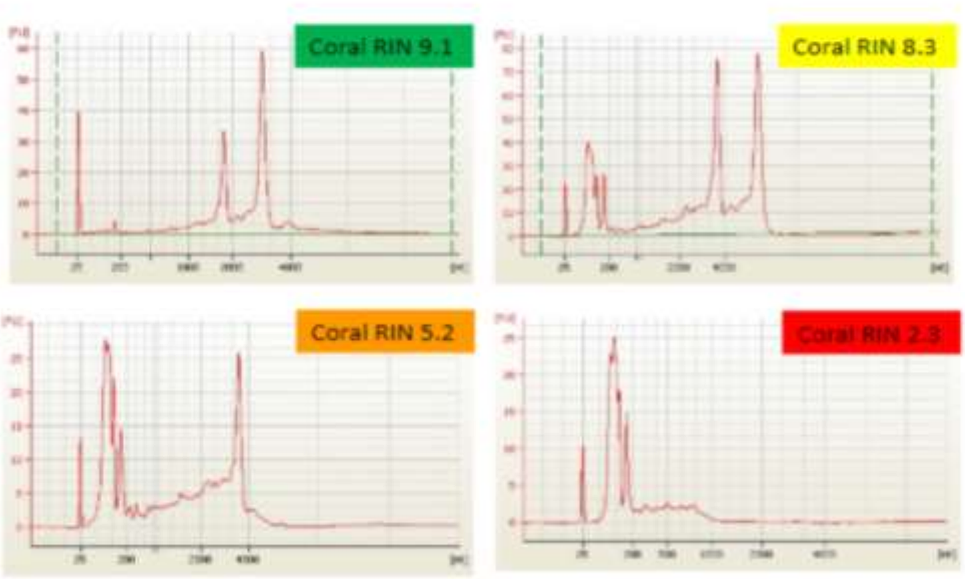

Supplementary Figure 6: Global view of the statistics generated by the control quality pipeline on a subset of sequences of each sequencing file. a) table containing the number of obtained sequences and the global quality score. b) plot of the Q30 score along the sequences. c) Nucleotides distribution along the reads (Read 1 and Read 2). d) Detection of the adapters used during the sequencing library process (Read 1 and Read 2). e) Table containing the statistics after quality trimming of the sequences. f) Tables containing the statistics after removal of rRNA reads in Meta and Dual Transcriptomics sequencing files. g) Estimation of the duplication rate. h) Table containing statistics after the merging process. The figure presents the distribution size of the merged sequences.

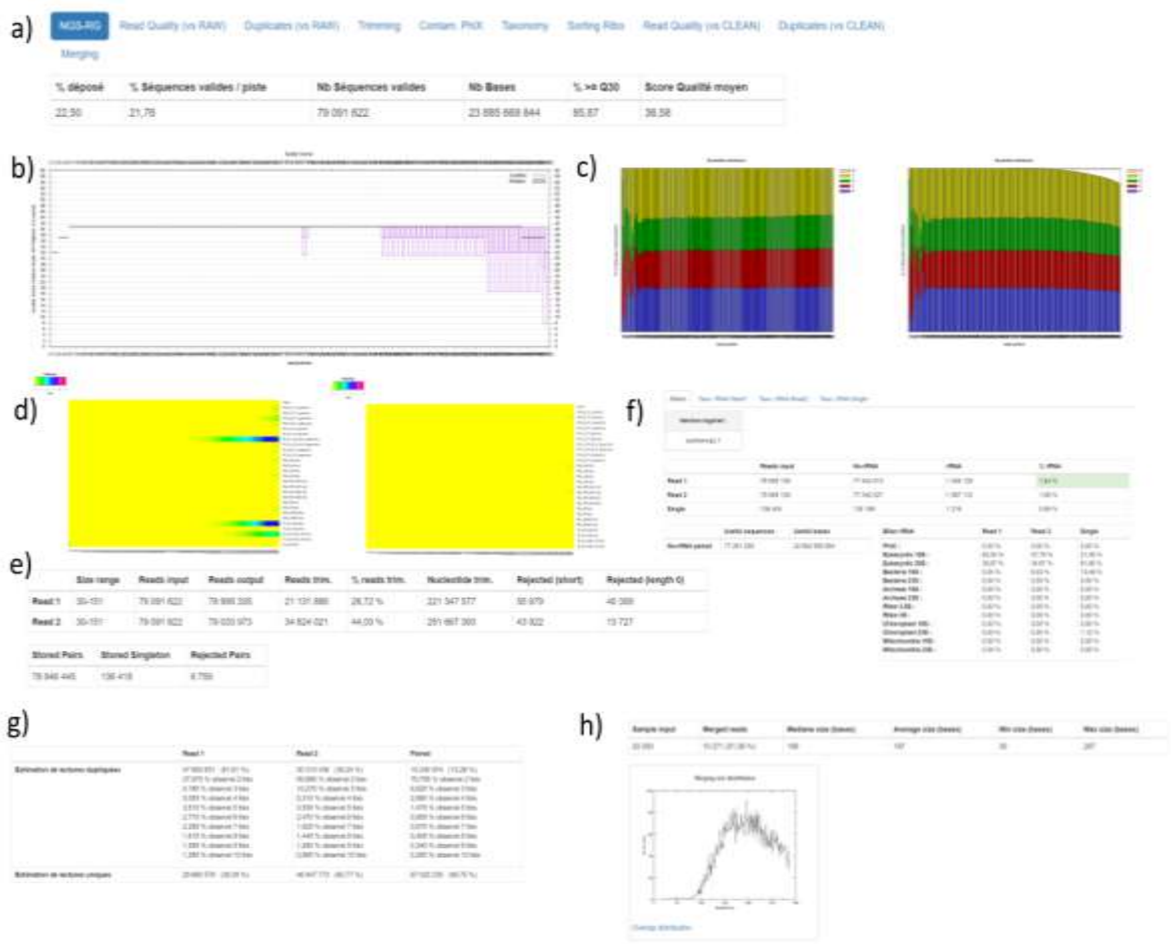

Supplementary Figure 7: Taxonomic assignment of a sequencing file. Taxonomic assignment was performed on a subset of reads from each sequencing dataset. Results allowed the validation of the sequencing files.

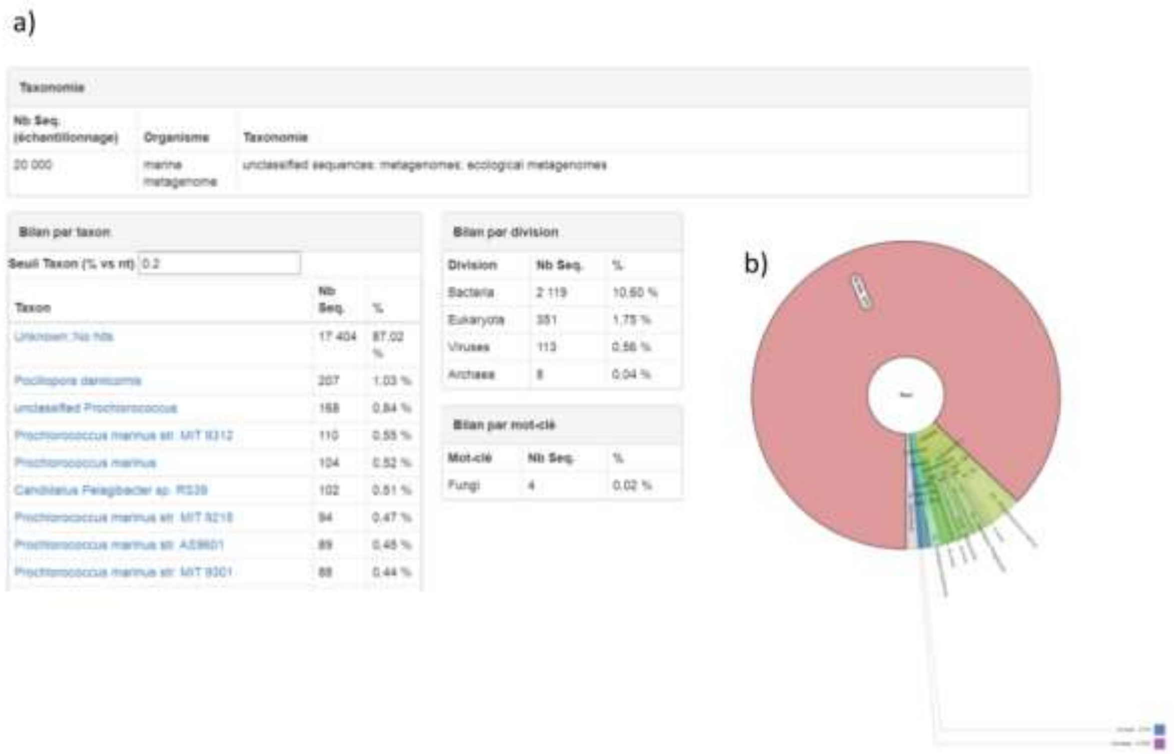

Supplementary Figure 8: Final report on the comparison between a metabarcoding sample and negative controls.

- PCR1

Cluster	OTU	Abundance	Abundance readset %	Abundance témoin %	Taxonomy	% id	% match length
BUW_AEVOOSTA_2_1_HHHYMDRXX.12BA218-BID02_clean.Cluster1	CEB_BABOSTA_2_1_HHHYMDRXX.12BA206-BID17.Cluster16	257 647	18.82	0.09	Eukaryota, Opisthokonta, Holozoa, Metazoa, Animalia, Craniata ...	100.00 %	100.00 %
BUW_AEVOOSTA_2_1_HHHYMDRXX.12BA218-BID02_clean.Cluster2	CEB_BABOSTA_2_1_HHHYMDRXX.12BA206-BID17.Cluster59	237 523	17.38	0.11	Eukaryota, Opisthokonta, Holozoa, Metazoa, Animalia, Craniata ...	100.00 %	100.00 %
BUW_AEVOOSTA_2_1_HHHYMDRXX.12BA218-BID02_clean.Cluster20	CEB_BABOSTA_2_1_HHHYMDRXX.12BA206-BID17.Cluster25	3 807	0.28	0.09	Bacteria, Proteobacteria, Alphaproteobacteria, Caulobacterales, Caulobacteraceae, uncultured	98.80 %	100.00 %
BUW_AEVOOSTA_2_1_HHHYMDRXX.12BA218-BID02_clean.Cluster51	CEB_BABOSTA_2_1_HHHYMDRXX.12BA206-BID17.Cluster114	3 459	0.25	0.05	Bacteria, Proteobacteria, Alphaproteobacteria, Caulobacterales, Caulobacteraceae, uncultured	99.20 %	100.00 %
BUW_AEVOOSTA_2_1_HHHYMDRXX.12BA218-BID02_clean.Cluster33	CEB_BABOSTA_2_1_HHHYMDRXX.12BA206-BID17.Cluster38	2 521	0.18	0.04	Bacteria, Proteobacteria, Gammaproteobacteria, Alteromonadales, Pseudoalteromonadaceae, Pseudoalteromonas	100.00 %	100.00 %

Supplementary Table 1. List of abbreviations used in this study.

Abreviation	Description
BID	Barcode IDentifier
BR	Broad Range
CDIV	Coral DIVersity
EMBL-EBI	EMBL European Bioinformatics Institute
ENA	European Nucleotide Archive
FFR	Fe-based virus Flocculation, Filtration, and Resuspension method
HS	High Sensitivity
LIMS	Laboratory Information Management System
NC	Negative Controls
NGL	Next Generation Laboratory Information Management System
NGL-BI	NGL-BioInformatics
NGL-P	NGL-Project management
NGL-S	NGL- Sample management
NGL-SQ	NGL- SeQuencing
NGL-SUB	NGL- SUBmission
NGS-QC	NGS- Quality Control
OTU	Operational Taxonomic Unit
R1, R2	Paired reads (Read 1, Read 2)
RIN	RNA Integrity Number
rRNA	ribosomal RNA
RTA	Illumina Real Time Analysis

Supplementary Table 2. Barcode Identifier (BID) sequences

BID name	Sequence	BID name	Sequence
**Bid01**	GTGTACAT	**Bid18**	CGAGTCGT
**Bid02**	TATGTCAG	**Bid19**	ACACACAC
**Bid03**	TAGTCGCA	**Bid20**	GTACGACT
**Bid04**	TACTATAC	**Bid21**	ATGATCGC
**Bid05**	ACTAGATC	**Bid22**	CATCAGTC
**Bid06**	GATCGCGA	**Bid23**	GATGATCT
**Bid07**	CGCTCTCG	**Bid24**	CTGCGTAC
**Bid08**	GTCGTAGA	**Bid25**	AGCGACTA
**Bid09**	GTCACGTC	**Bid26**	TCAGTGTC
**Bid10**	GCGTCAGC	**Bid27**	CTATGCTA
**Bid11**	TGACATCA	**Bid28**	TCGCGCTG
**Bid12**	ACATGTGT	**Bid29**	AGCACAGT
**Bid13**	AGACTATG	**Bid30**	TAGCTAGT
**Bid14**	ACGACGAG	**Bid31**	AGTGCTAC
**Bid15**	TCTACTGA	**Bid32**	CGTATACA
**Bid16**	ACTCTGCT	**Bid33**	CACATGAT
**Bid17**	ATATAGCG		

Supplementary Table 3: PCR mixtures for Metabarcoding experiments.

	Finnzyme Phusion® High-Fidelity PCR Master Mix with GC Buffer				
**Input**	**DNA < 1 ng/µl**	**DNA 1 to 5 ng/µl**	**DNA > 5 ng/µl**	**Positive control**	**Negative control**
**DNA normalization**	**none**	**1 ng/µl**	**5 ng/µl**	**5 ng/µl**	**none**
DNA input (µl)	10	10	2	2	0
Mix Phusion 2X (µl)	12.5	12.5	12.5	12.5	12.5
Primers Forward 10 µM (µl)	1	1	1	1	1
Primers Reverse 10 µM (µl)	1	1	1	1	1
DMSO (µl)	0.75	0.75	0.75	0.75	0.75
H2O Ambion (µl)	0	0	7.75	7.75	10
Total volume (µl)	25.25	25.25	25	25	25.25

	QIAGEN Multiplex PCR Kit				
**Input**	**DNA < 1 ng/µl**	**DNA 1 to 5 ng/µl**	**DNA > 5 ng/µl**	**Positive control**	**Negative control**
**DNA normalization**	**none**	**1 ng/µl**	**5 ng/µl**	**5 ng/µl**	**none**
DNA input (µl)	3	3	2	2	0
2x QIAGEN Multiplex PCR Master Mix (µl)	12.5	12.5	12.5	12.5	12.5
Primer F 2.5 µM (µl)	2	2	2	2	2
Primer R 2.5 µM (µl)	2	2	2	2	2
H2O Ambion (µl)	5.5	5.5	6.5	6.5	8.5
Total volume (µl)	25	25	25	25	25

	Bioline, Mytaq HS kit		
**Input**	**DNA sample**	**Positive control**	**Negative control**
DNA input (µl)	4	1	0
MyTaq HS Mix , 2x (µl)	12.5	12.5	12.5
Primer F 10 µM (µl)	0.5	0.5	0.5
Primer R 10 µM (µl)	0.5	0.5	0.5
H2O Ambion (µl)	7.5	10.5	11.5
Total volume (µl)	25	25	25

Supplementary Table 4: PCR amplification cycling protocols for metabarcoding experiments.

Protocol	primers name	primers sequences	expected size	Polymerase	Thermocycling			Ampure beads Volume for Purification
					Temperature	Time	Cycle Nb	
16SV4V5	515F<br>926R	5′-GTGYCAGCMGCCGCGGTAA-3′<br>5′-CCGYCAATTYMTTTRAGTTT-3′	411 bp for Bacteria, Archaea amplification<br>600 bp for eukaryote amplification	Phusion High-Fidelity GC Master Mix	98°C	30 sec		1V
					98°C	10 sec	25 cycles	
					53°C	30 sec		
					72°C	30 sec		
					72°C	10 min		
					4°C	∞		
				PCR amplification Master Mix	95°C	30 sec		
					94°C	10 sec	30 cycles	
					53°C	60 sec		
					72°C	30 sec		
					72°C	10 min		
					4°C	∞		
				Mytaq HS mix	95°C	60 sec		
					95°C	15 sec	35 cycles	
					55°C	15 sec		
					72°C	10 sec		
					72°C	10 min		

					4°C	∞		
16S V4V5 NESTED PCR<br><br>16S Full Length<br><br>±<br><br>16SV4V5	27F<br><br>1492R	5'-AGAGTTTGATCMTGGCTCAG–3′<br><br>5’-TACGGYTACCTTGTTACGACTT–3′	1400 bp	Phusion High-Fidelity GC Master Mix	98°C	5 min		1V
					98°C	30 sec	20 cycles	
					55°C	30 sec		
					72°C	60 sec		
					72°C	10 min		
					4°C	∞		
	515F<br><br>926R	5′-GTGYCAGCMGCCGCGGTAA-3′<br><br>5′-CCGYCAATTYMTTTRAGTTT-3′	411 bp for Bacteria, Archaea amplification<br><br>600 bp for eukaryote amplification		98°C	30 sec		1V
					98°C	10 sec	25 cycles	
					53°C	30 sec		
					72°C	30 sec		
					72°C	10 min		
					4°C	∞		
18SV9	1389F<br><br>1510R	5′-TTGTACACACCGCCC-3′<br><br>5′-CCTTCYGCAGGTTCACCTAC-3′	150-170 bp	Phusion High-Fidelity GC Master Mix	98°C	30 sec		1.8V
					98°C	10 sec	25 cycles	
					57°C	30 sec		
					72°C	30 sec		
					72°C	10 min		
					4°C	∞		
				PCR amplification Master Mix	95°C	15 min		1.8V
					94°C	30 sec	30 cycles	

					57°C	60 sec		
					72°C	30 sec		
					72°C	10 min		
					4°C	∞		
ITS2 Symbiodiniaceae	SYM_VAR_5.8S2<br>SYM_VAR_REV	5’-GAATTGCAGAACTCCGTGAACC-3’<br>5’-CGGGTTCWCTTGTYTGACTTCATGC-3’	300 bp	Phusion High-Fidelity GC Master Mix	98°C	2 min		1V
					98°C	30 sec	30 cycles	
					56°C	30 sec		
					72°C	30 sec		
					72°C	5 min		
					4°C	∞		
			300 bp	PCR amplification Master Mix	95°C	15 min		1V
					94°C	30 sec	35 cycles	
					56°C	60 sec		
					72°C	90 sec		
					72°C	10 min		
					4°C	∞		
